# Supplementary material for: AI-embodied multi-modal flexible electronic robots with programmable sensing, actuating and self-learning
Source: Nat Commun. 2025 Oct 3;16:8818. doi: 10.1038/s41467-025-63881-6 (PMC12494961; doi:10.1038/s41467-025-63881-6)
Supplement: Supplementary file 1 — Supplementary Information [file 41467_2025_63881_MOESM1_ESM.pdf]

Supplementary Materials for

AI-embodied multi-modal flexible electronic robots with programmable sensing, actuating and self-learning

Junfeng Li[1], Zhangyu Xu[2], Nanpei Li[1], Kaijun Zhang[3], Guangyong Xiong[1], Minjie Sun[1], Chao Hou[2], Jingjing Ji[2], Fan Zhang[2*], Junwen Zhong[3*], and YongAn Huang[2*]

Affiliations

[1]School of Mechanical and Electronic Engineering, Wuhan University of Technology, Wuhan 430070, China.

[2]State Key Laboratory of Intelligent Manufacturing Equipment and Technology, Huazhong University of Science and Technology, Wuhan 430074, China.

[3]Department of Electromechanical Engineering, Centre for Artificial Intelligence and Robotics, University of Macau, Macau SAR 999078, China.

* Corresponding authors:

fanzhang@hust.edu.cn

junwenzhong@um.edu.mo

yahuang@hust.edu.cn

The PDF file includes:

Note 1: Output force measurement of the oscillator.

Note 2: Simulation and experimental results for the detailed locomotion of FEbot.

Note 3: Calibration of strain sensor.

Note 4: Shape reconstruction method.

List of symbols and abbreviations

τ_o	oscillation frequency
δ_O	constant of electromotive force
ϕ_O	magnetic flux
U_O	input voltage of oscillator
I_O	input current of oscillator
r_O	resistance of oscillator
F_x	input force of oscillator in X direction
F_y	input force of oscillator in Y direction
m_F	mass of FEbot
g	gravitational acceleration
x	displacement in X direction
$\dot{x}$	velocities of center of gravity in X direction
$\ddot{x}$	accelerations of center of gravity in X direction
y	displacement in Y direction
$\ddot{y}$	accelerations of center of gravity in Y direction
t	time
t_1, t_2, t_3	time in state I, II, III
F_N	normal force exerted on setae
F_{ni}	normal force exerted on i-th seta
F_{f-}	magnitude of the friction force that opposes the backward sliding motion
F_{f+}	magnitude of the friction force that opposes the forward sliding motion
T_U	cycle time
k_i	stiffness of i-th seta in Y direction
υ_i	damping coefficient of i-th seta in Y direction
$k_i^{'}$	stiffness of i-th seta in X direction
$\upsilon_i^{'}$	damping coefficient of i-th seta in X direction
$\mu(\mu_-, \mu_+)$	positive friction coefficients
μ_-	friction coefficient for F_{f-}
μ_+	friction coefficient for F_{f+}
β	angle of inclined surface
l	length of inclined surface
h	height of inclined surface
P	applied load

C_G	mass center
H, H_1	height of the FEbot with and without load
Δs_f	forward displacement
Δs_b	backward displacement
Δs_h	highest position
Δs_l	lowest position
L	seta length
d	the diameter of the seta
θ	the contact angle between the seta and the ground
s	reference arclength
$\mathbf{p}$	global position in Cartesian coordinates
$\mathbf{R}$	rotation matrix of material orientation
$\mathbf{n}$	internal force in the global frame
$\mathbf{m}$	internal moment in the global frame
$\mathbf{f}$	distributed force in the global frame
$\bar{\mathrm{f}}$	encompasses any residual forces not explicitly modeled
$\mathbf{l}$	distributed moment in the global frame
$\mathbf{v}$	rate of change of position with respect to arclength in the local frame
$\mathbf{u}$	curvature vector in the local frame
$\mathbf{q}$	Speed in the local frame
$\boldsymbol{\omega}$	angular velocity in the local frame
E	young's modulus of seta
G	shear modulus of seta
ρ	material density
A	cross-sectional area
$\mathbf{J}$	second mass moment of inertia tensor
$\mathbf{K_{se}}$	stiffness matrix for shear and extension
$\mathbf{K_{bt}}$	stiffness matrix for bending and twisting
$\mathbf{B_{se}}$	damping matrix for shear and extension
$\mathbf{B_{bt}}$	damping matrix for bending and twisting
$\mathbf{C}$	square law drag coefficient matrix
ε	strain of sensor
l_0	initial length of sensor
l_1	elongated length of sensor
V_{out}	output voltage between Pin 1 and 3
V_{in}	input voltage between Pin 2 and 4
r_z	resistance of sensor, subscript $z = 1,2,3,4$ denotes the pin number
δ	thickness of infinitesimal element
Q	length of infinitesimal element
ΔQ	variation length after deformation
χ	length of central layer
ϑ	bending angle
φ_ς	curvature of ς-th segment, ς denotes the segment number

ε_ς	strain of ς-th segment
P_ς	the origin of the coordinate system $M_\varsigma(a_\varsigma, b_\varsigma)$
α_ς	angle of between $P_\varsigma P_{\varsigma+1}$ and b_ς
ξ	number of segments
Φ	distance between center of adjacent sensors
ds	length of arbitrary segment
M_ς	local coordinate system
$\Psi_{\varsigma+1}$	transformation matrix from local coordinate system M_ς to $M_{\varsigma+1}$
Γ	global coordinate system
Λ	transformation matrix from $M_{\varsigma+1}$ to Γ
η	arc radius
κ	curvature
Z_j	time at jth moment
φ_{1j} , $\varphi_{2j}, \varphi_{3j}$	curvature of three sensors at j-th moment
$\varkappa_1$, $\varkappa_2$, $\varkappa_3$	curvature matrix for three sensors from time Z_0 to Z_{j+1}
v_{Febot}	average moving speed of FEbot
HVE	the function that codes the features into hyperdimensional vectors
Ham	the Hamming distance
H_{HV}	the encoded HVs.
f_{TD}	the time domain TD features of the raw data
$\mathbf{W}_i$	the i-th categorized HV
D	the dimension of the HV
$\mathbf{E}_j$	the encoded HV query of the j-th test sample
pwm_{set}	the control signal determined by the robot posture according to the Hamming distance
PWM_z	the z-th oscillator's value of PWM.

Table 1 Seta Materials Comparison

Reference	**Materials**	**Mechanical Properties**	**Durability**	**Environmental Adaptability**
[21], [23-25],	**Polydimethylsiloxane (PDMS)/Silicone**	**Advantages**: Low elastic modulus (0.1-5 MPa), high softness and deformability<br>**Limitations:** Prone to plastic deformation under large strains (e.g., permanent compression in silicone)	**Limitations:** UV/ozone-induced aging	**Limitations:** Thermal degradation (silicone softens >200°C,<br>PDMS decomposes >300°C)
[22]	**Resin**	**Advantages:** Tunable elastic modulus (1-10 GPa), photocurable resins enable precise complex structures.<br>**Limitations:** High brittleness, susceptibility to cracking	**Limitations:** Low stress fatigue threshold (cracking at <10^4 cycles)	**Limitations:** High thermal expansion coefficient (50-100 ppm/°C), solvent-induced swelling
**This work**	**SSMA**	**Advantages:** Exceptional elastic deformation capacity, high elastic modulus (~30-80 GPa), suitable for high-load applications<br>**Limitations:** High rigidity	**Advantages:** Long fatigue life (10^6-10^7 cycles), wear resistance, no aging issues.<br>**Limitations:** Phase transformation accumulation under cyclic loads	**Advantages:** Broad temperature tolerance (-200°C to 400°C), corrosion resistance

Table 2 Comparison of Small-Scale Soft Robots

Reference	Adaptability to environment					Vertical substrate speed (BL/s)	Perceiving Information	Embodied AI	Variable structure	Load-bearing
	Smooth ground	Rough ground	Stair	Under water	Vertical substrate					
[3]	Yes	Yes	Yes	Yes	Yes	0.008	-	No	No	No
[10]	Yes	Yes	No	No	Yes	0.75	Vison	No	No	No
[11]	Yes	No	Yes	No	No	-	Gas	No	No	No
[26]	Yes	Yes	Yes	Yes	Yes	0.028	-	No	No	36 g
[27]	Yes	Yes	No	No	No	-	-	No	No	59.5 kg
[28]	Yes	Yes	No	No	No	-	-	No	No	No
[29]	Yes	Yes	No	No	No	-	-	No	No	No
[30]	Yes	Yes	No	No	No	-	Vison	No	No	flattened by a fly swatter
[31]	Yes	Yes	No	Yes	Yes	0.004	-	No	No	No
[32]	Yes	No	No	No	Yes	1.4	Vison	No	No	No
This work	Yes	Yes	Yes	Yes	Yes	2.3	Posture, strain, temperature, humidity, proximity, vison	Yes	Yes	500 kg

Note 1: Output force measurement of the oscillator

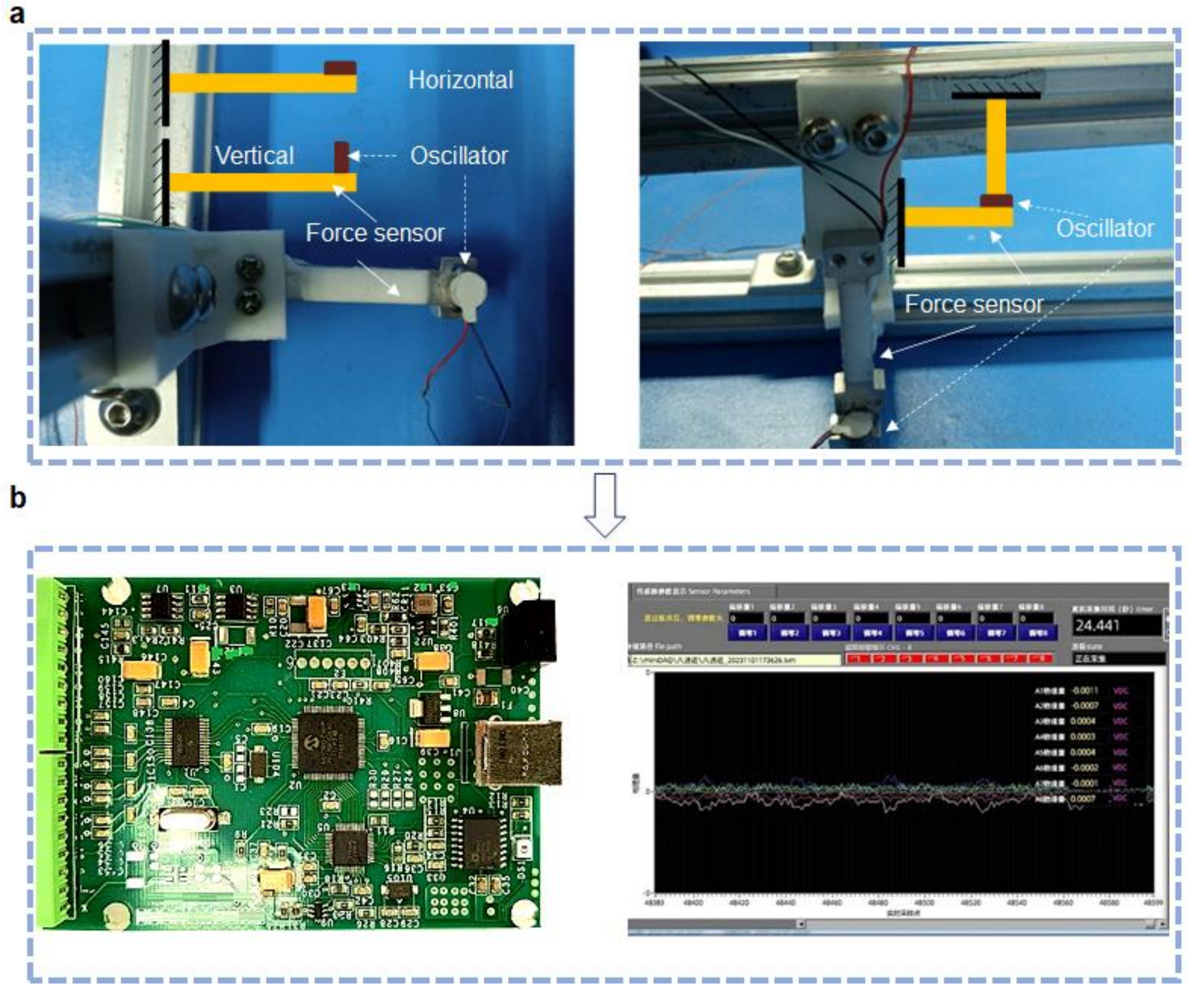

Fig. 1. Experimental setup for measuring the oscillator's output force. **(a)** Force measurement using two synchronized sensors. **(b)** AUMANYU four-channel data acquisition card and software interface (http://www.aumanyu.com/about/).

The FEbot comprises a 22 mm-long film, supporting one oscillator and four setae (fabricated from superelastic shape-memory alloy, SSMA) oriented at a 45° inclined angle. The oscillator—a coin-type coreless vibration motor—generates periodic vibrations when a DC voltage of 3 to 5 V is applied. Its frequency is defined as:

$$\tau_o = \frac{\delta_o \phi_o}{U_o - I_o r_o} \tag{1}$$

where δ_o is the constant of electromotive force, ϕ_o is the magnetic flux; U_o, I_o and r_o denote the oscillator's input voltage, current, and resistance, respectively. The oscillator's output force is measured using force sensors and a data acquisition system, as depicted in **Fig.**

1. The oscillator is placed in horizontal and vertical directions to obtain the magnitude of force in X and Y directions, respectively. Phase difference is quantified by simultaneously recording output forces with two synchronized sensors (**Fig. 1a**). A four-channel isolated 24-bit data acquisition card (AUMANYU), featuring high speed, resolution, precision, and ultra-low noise, is employed for phase measurements (**Fig. 1b**).

The output force is measured when the oscillator is driven by different voltages (3–5 V, **Figs. 2a–2c**). Experimental data demonstrate that as the voltage increases from 3 to 5 V, the X-direction output force rises from 0.113 to 0.381 N, while the frequency increases from 260 to 450 Hz; conversely, the Y-direction output force remains nearly constant (**Fig. 2d**).

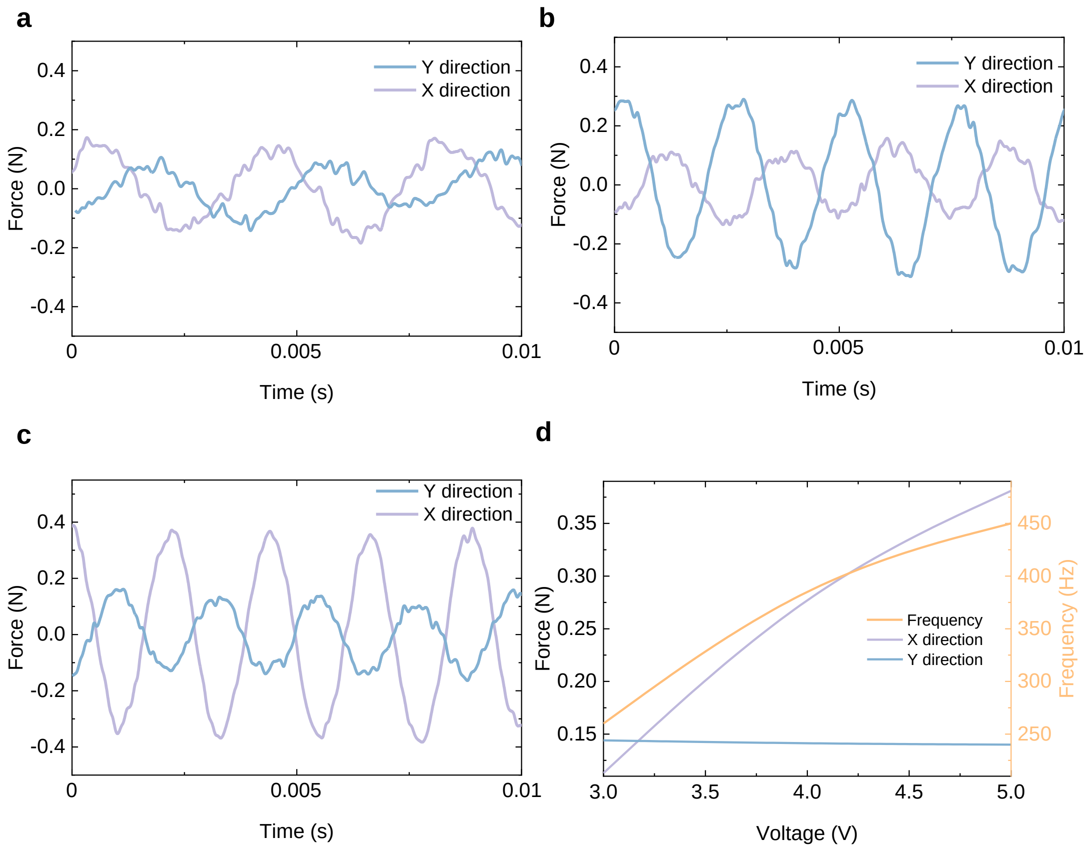

Fig. 2. Oscillator output force characterization. (**a–c**) Measured X- and Y-direction forces under applied voltages of 3, 4, and 5 V, respectively. (**d**) Correlation between force amplitude, driving frequency, and applied voltage (3 to 5 V).

Note 2: Simulation and experimental results for the detailed locomotion of FEbot

1. Dynamic models

The dynamic model of the FEbot is derived as follows (**Fig. 3a**). Assuming the motor's force is uniformly transmitted to each seta, the system is modeled as a classical spring-damper system, a standard approach for vibratory robotic motion. The robot's mass is lumped at the geometric center C_G of the driver, located at coordinates (x, y). The governing equations are:

$$\begin{cases} F_x - F_{f+} - \sum_i^4 (k_i^{'} x_b + v_i^{'} \dot{x}_b) = m_F \ddot{x} & \dot{x} > 0 \\ F_x - F_{f-} - \sum_i^4 (k_i^{'} x_b + v_i^{'} \dot{x}_b) = m_F \ddot{x} & \dot{x} \le 0 \end{cases} \quad i = 1,2,3,4 \tag{2}$$

$$\begin{cases} F_y + mg - F_N = m_F \ddot{y} \\ F_N = \sum_i^4 F_{ni} \\ F_{ni} = k_i y + v_i \dot{y} \end{cases} \quad i = 1,2,3,4 \tag{3}$$

where F_x and F_y are the oscillator's input forces in the X- and Y-directions, respectively; m is the FEbot's mass; g is gravitational acceleration; x and y are displacements in the X- and Y-directions; F_{f+} and F_{f-}denote the friction forces opposing forward and backward sliding motions, respectively; $k_i^{'}$ and $v_i^{'}$ are the stiffness and damping coefficients in the X-direction for the i-th seta; F_{ni} is the normal contact force of the i-th seta; F_N is the total normal contact force between the FEbot and substrate; k_i and v_i are the stiffness and damping coefficients in the Y-direction; x_b and $\dot{x}_b$ represent the displacement and velocity of the seta tip relative to point O_1 in the X_1-direction of the $X_1O_1Y_1$ coordinate system.

As shown in **Fig. 3(b)**, x_b and $\dot{x}_b$ can be obtained by using Cosserat rod theory which is used to model the bending shape of each seta [45].

$$\begin{cases} \mathbf{p}_s = \mathbf{R}\mathbf{v} \\ \mathbf{p}_t = \mathbf{R}\mathbf{q} \\ \mathbf{R}_s = \mathbf{R}\hat{\mathbf{u}} \\ \mathbf{R}_t = \mathbf{R}\hat{\boldsymbol{\omega}} \\ \mathbf{n}_s = \rho A \mathbf{R}(\hat{\boldsymbol{\omega}}\mathbf{q} + \mathbf{q}_t) - \mathbf{f} \\ \mathbf{m}_s = \partial_t(\mathbf{R}\rho\mathbf{J}\boldsymbol{\omega}) - \hat{\mathbf{p}}_s\mathbf{n} - \mathbf{l} \\ \mathbf{q}_s = \mathbf{v}_t - \hat{\mathbf{u}}\mathbf{q} + \hat{\boldsymbol{\omega}}\mathbf{v} \\ \boldsymbol{\omega}_s = \mathbf{u}_t - \hat{\mathbf{u}}\boldsymbol{\omega} \end{cases} \tag{4}$$

where **p** denotes the global position; **R** represents the rotation matrix; **v** is the rate of change of position with respect to arclength in the local frame; **q** and **ω** are the linear velocity and angular velocity in the local coordinate system, respectively; **u** is the curvature vector in the local frame; **n** is the internal force; **m** is the internal moment; ^ indicates the conversion of

the variables into a skew-symmetric matrix. $\mathbf{f}$ is the distributed force in the global frame; ρ is material density; A is the cross-sectional area; $\mathbf{J}$ is the second mass moment of inertia tensor.

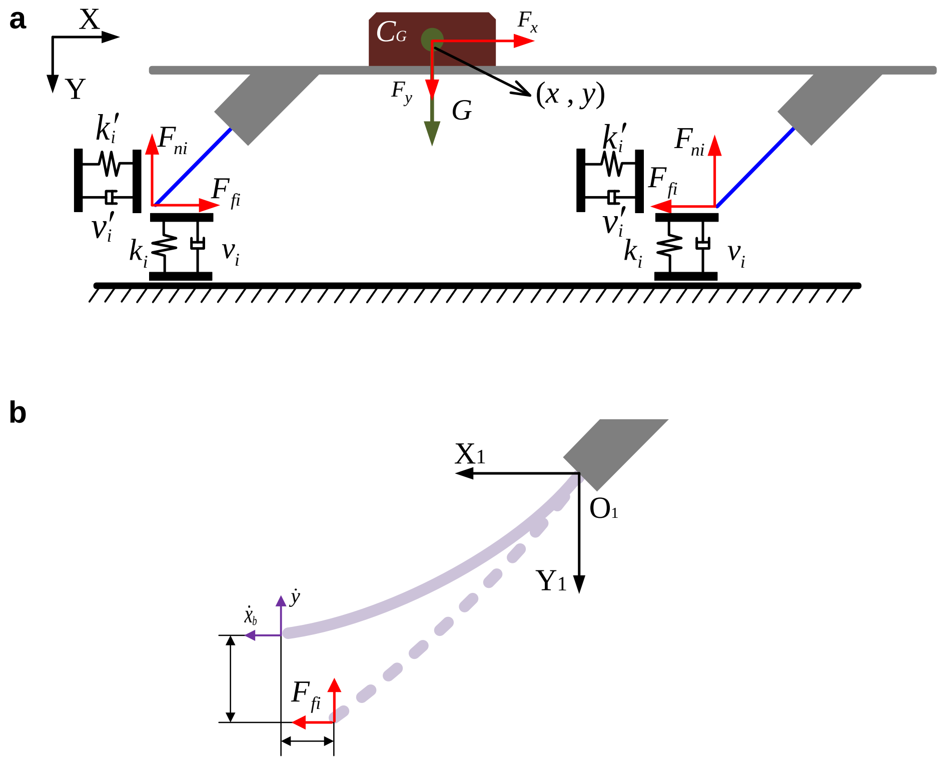

Fig. 3. Simplified dynamic model of the FEbot. **(a)** Spring-damper system representation. **(b)** Seta force analysis based on Cosserat theory.

The moment of inertia can be expanded as:

$$\partial_t(\mathbf{R}\rho\mathbf{J}\boldsymbol{\omega}) = \rho\mathbf{R}(\hat{\boldsymbol{\omega}}\mathbf{J}\boldsymbol{\omega} + \mathbf{J}\boldsymbol{\omega}_t) \tag{5}$$

Considering the self-weight of the bristle and the aerodynamic resistance, the distributed force f can be expressed as:

$$f = \rho A g - \mathbf{RCq} \odot |\mathbf{q}| + \bar{f} \tag{6}$$

where $\bar{f}$ encompasses any residual forces not explicitly modeled, and the symbol$\odot$ denotes the Hadamard product (element-wise multiplication):

$$\mathbf{q} \odot |\mathbf{q}| = [q_1^2 \operatorname{sgn}(q_1) \quad q_2^2 \operatorname{sgn}(q_2) \quad q_3^2 \operatorname{sgn}(q_3)]^T \tag{7}$$

From the material constitutive law, we have:

$$\begin{cases} \mathbf{n} = \mathbf{R}[\mathbf{K_{se}}(\mathbf{v} - \mathbf{v}^*) + \mathbf{B_{se}}\mathbf{v}_t] \\ \mathbf{m} = \mathbf{R}[\mathbf{K_{bt}}(\mathbf{u} - \mathbf{u}^*) + \mathbf{B_{bt}}\mathbf{u}_t] \end{cases} \tag{8}$$

For the partial differential equation (PDE) system (4), a specific implicit differentiation

scheme can be applied to discretize the time derivative terms, thereby reducing the problem to a spatial ordinary differential equation (ODE). For discretized variables, the superscript notation is used to denote the time step index. A general first-order implicit differentiation formula can be uniformly written in the following form:

$$ {}^{(i)}y_t \approx c_0\,{}^{(i)}y + \sum_{k=1}^{\infty}[c_k\,{}^{(i-k)}y + d_k\,{}^{(i-k)}y_t] := c_0\,{}^{(i)}y + {}^{(i)}\overset{h}{y} \tag{9} $$

Among them, ${}^{(i)}\overset{h}{y}$ represents the sum of all remaining terms dependent on the historical values of y. In this paper, the backward differentiation formulas (BDF)-α method is employed to solve the system, which can be expressed as:

$$ {}^{(i)}y_t = c_0\,{}^{(i)}y + c_1\,{}^{(i-1)}y + c_2\,{}^{(i-2)}y + d_1\,{}^{(i-1)}y_t \tag{10} $$

where:

$$ \begin{cases} c_0 = (1.5+\alpha)/[\delta t(1+\varrho)] \\ c_1 = -2/\delta t \\ c_2 = (0.5+\varrho)/[\delta t(1+\varrho)] \\ d_1 = \alpha/(1+\varrho) \end{cases} \tag{11} $$

Using this method, after discretizing the time derivatives, the distributed forces and moments of inertia are substituted into Eq. (4). The partial differential equations are thereby simplified into a system of ordinary differential equations with respect to arc length:

$$ \begin{cases} \mathbf{p}_s = \mathbf{R}\mathbf{v} \\ \mathbf{R}_s = \mathbf{R}\hat{\mathbf{u}} \\ \mathbf{n}_s = \mathbf{R}[\rho A(\hat{\boldsymbol{\omega}}\mathbf{q} + \mathbf{q}_t) + C\mathbf{q} \odot |\mathbf{q}|] - \rho A g - \bar{\mathbf{f}} \\ \mathbf{m}_s = \rho\mathbf{R}(\hat{\boldsymbol{\omega}}\mathbf{J}\boldsymbol{\omega} + \mathbf{J}\boldsymbol{\omega}_t) - \hat{\mathbf{p}}_s\mathbf{n} - \mathbf{l} \\ \mathbf{q}_s = \mathbf{v}_t - \hat{\mathbf{u}}\mathbf{q} + \hat{\boldsymbol{\omega}}\mathbf{v} \\ \boldsymbol{\omega}_s = \mathbf{u}_t - \hat{\mathbf{u}}\boldsymbol{\omega} \end{cases} \tag{12} $$

The linear constitutive relationship can be written as:

$$ \begin{cases} \mathbf{v} = (\mathbf{K_{se}} + c_0\mathbf{B_{se}})^{-1}(\mathbf{R}^{\mathrm{T}}\mathbf{n} + \mathbf{K_{se}}\mathbf{v}^* - \mathbf{B_{se}}\overset{h}{\mathbf{v}}) \\ \mathbf{u} = (\mathbf{K_{bt}} + c_0\mathbf{B_{bt}})^{-1}(\mathbf{R}^{\mathrm{T}}\mathbf{m} + \mathbf{K_{bt}}\mathbf{u}^* - \mathbf{B_{bt}}\overset{h}{\mathbf{u}}) \end{cases} \tag{13} $$

All time derivative terms in Eq. (13) are computed from the state variables at the current or previous time steps using the following equation:

$$ \begin{cases} \mathbf{v}_t = c_0\mathbf{v} + \overset{h}{\mathbf{v}} \\ \mathbf{u}_t = c_0\mathbf{u} + \overset{h}{\mathbf{u}} \\ \mathbf{q}_t = c_0\mathbf{q} + \overset{h}{\mathbf{q}} \\ \boldsymbol{\omega}_t = c_0\boldsymbol{\omega} + \overset{h}{\boldsymbol{\omega}} \end{cases} \tag{14} $$

By applying the boundary condition of the force $\mathbf{n}_L(t) = [F_{ni}(t), F_{fi}(t)]$, $\mathbf{m}_L(t) = 0$ acting on the bristle tip and solving the ODE system in Eqs. (12) to (14), x_b and $\dot{x}_b$ can be obtained according to the following equation:

$$\begin{cases} \mathbf{p}_x = f(\mathbf{p}_y) = 0.7592\mathbf{p}_y + 0.0488 \\ \mathbf{q}_x = g(\mathbf{q}_y) = -0.7379\mathbf{q}_y \\ \mathbf{p}_x = x_b, \mathbf{p}_y = y\,, \mathbf{q}_x = \dot{x}_b, \mathbf{q}_y = \dot{y} \end{cases} \quad (15)$$

Finally, the displacement x and y in X and Y direction can be obtained, respectively.

2. Solution approaches to the dynamical models

According to the experimental results shown in **Fig. 4a**, the input force for the simulation can be expressed by:

$$F_x = 0.142 \sin(1632t) \quad (16)$$

$$F_y = 0.097 \sin\big(1632(t - 0.0014)\big) + 0.002079 \quad (17)$$

To accurate model the locomotion, the force in Y direction needs to be modified as:

$$F_y = \gamma\big\{0.097 \sin[1632(t - 0.0014)]\big(1632(t - 0.0014)\big) + 0.002079\big\} \quad (18)$$

where γ=1.4 is the coefficient of correction.

The FEbot demonstrates vibration in Y direction when moving forward. The stiffness of setae in Y direction needs to be measured as well. As shown in **Fig. 4b**, the stiffness k_i is expressed by:

$$k_i = \frac{Pg}{4(H - H_1)}, \quad i = 1,2,3,4 \quad (19)$$

where P is the applied load; H and H_1 are the height of the FEbot with and without load, respectively.

The normal contact force of each seta F_{ni} according to Eq. (3, 18) can be used to calculate x_b and $\dot{x}_b$. To simplify the simulation method, the friction between substrate and each seta F_{fi} is expressed as:

$$F_{fi} = \gamma_2 F_{ni}. \quad (20)$$

where γ_2=0.18 is the coefficient of correction.

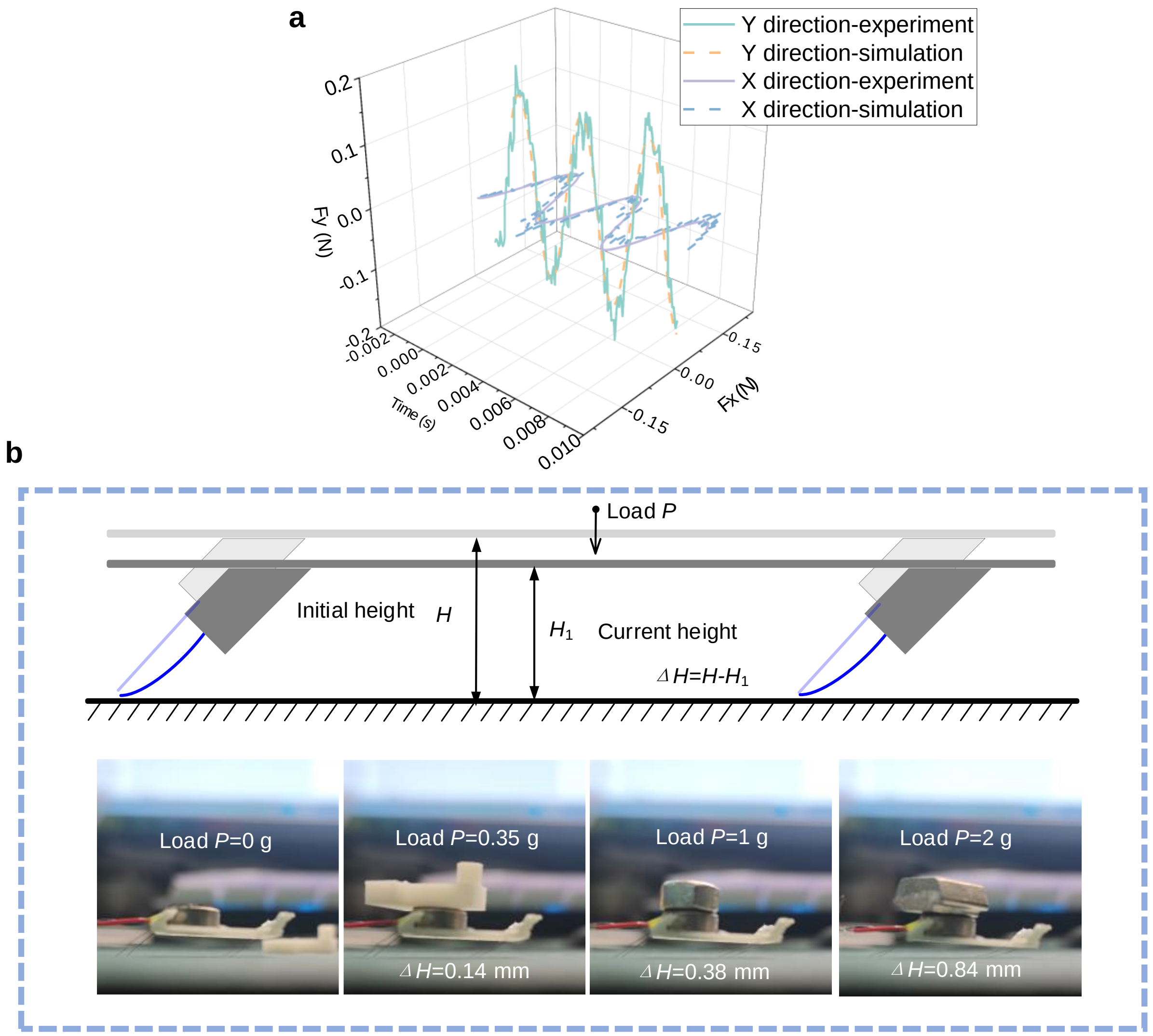

Fig. 4. Input force and stiffness identification. **(a)** Simulated input force calibrated against experimental data (Fig. 2c). **(b)** Seta stiffness measurement: average stiffness = 24.5 N/m (individual measurements: 2.5, 2.63, and 2.38 N/mm).

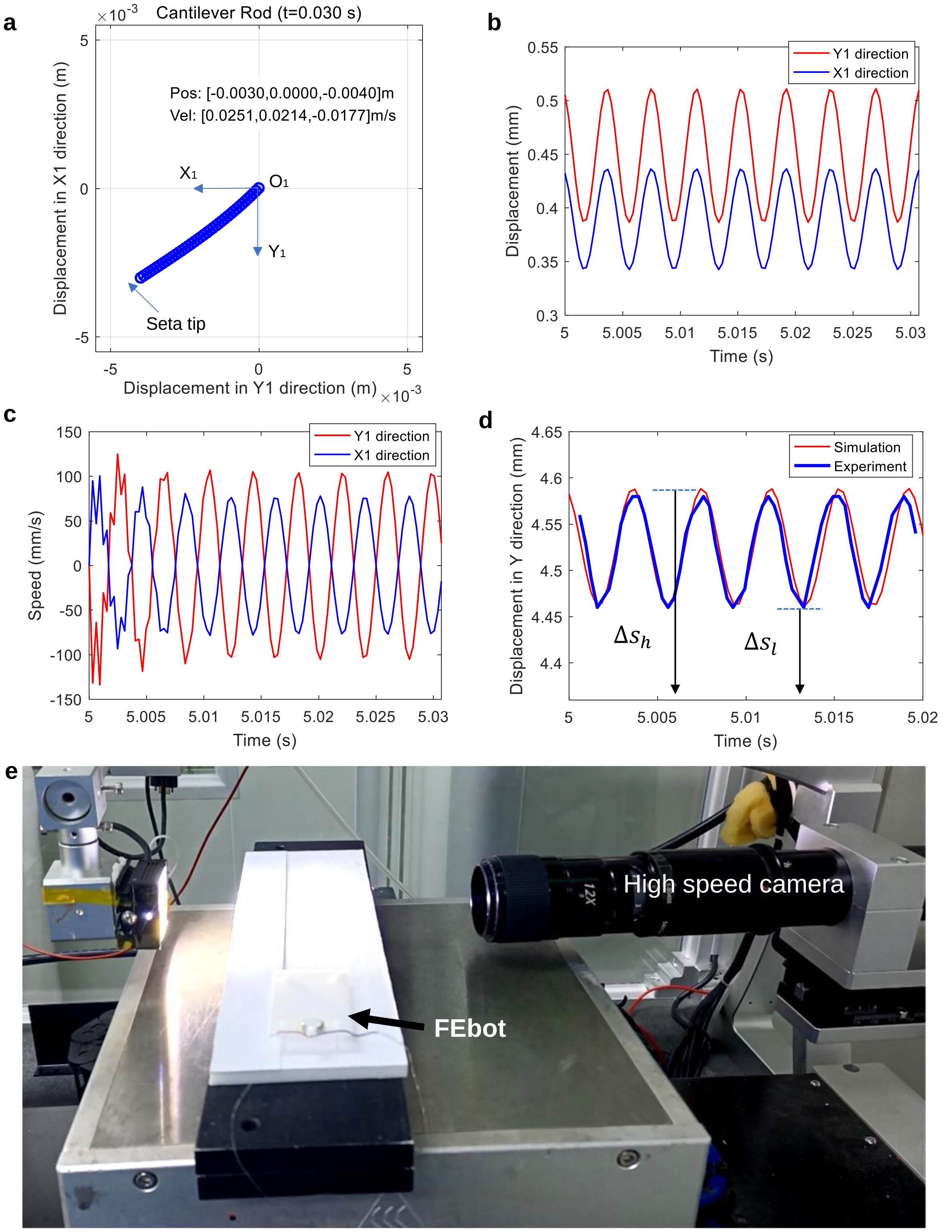

Fig. 5. Seta deformation based on the Cosserat model. (a) Time-resolved bending process. **(b)** Tip displacement (x_b in X_1 direction and y in Y_1 direction). **(c)** Tip velocity ($\dot{x}_b$ in X_1 direction and $\dot{y}$ in Y_1 direction). **(d)** Five-cycle Y-direction displacement: simulation vs. high-speed camera data Supplementary **Movie 1**). **(e)** High-speed camera experimental setup.

Fig. 5a illustrates the bending process of the seta over time. **Fig. 5b-c** present the simulation results of the tip's displacement (x_b in X_1 direction and y in Y_1 direction) and speed ($\dot{x}_b$ in X_1 direction and $\dot{y}$ in Y_1 direction) relative to point O1 in the $X_1O_1Y_1$ coordinate system, respectively. The Y_1-direction displacement in **Fig. 5b** is transformed into the vertical amplitude in the global Y-direction, which agrees qualitatively with experimental observations. Thus, x_b and $\dot{x}_b$ are assumed to be accurately obtained and utilized in Eq. (2) to calculate the displacement x in the X -direction.

The friction in Eq. (2) can be expressed by:

$$\begin{cases} F_{f-} = \mu_- F_N \\ F_{f+} = \mu_+ F_N \end{cases} \tag{S21}$$

where μ_- and μ_+ are positive friction coefficients satisfying $\mu_- \gg \mu_+$. These coefficients $\mu(\mu_-, \mu_+)$ are measured via the method depicted in **Fig. 3**. The FEbot is placed on an inclined surface, and the sliding velocity varies with the inclination angle β. Under uniform sliding motion, the equilibrium equations yield:

$$\begin{cases} mg\sin\beta = \mu F_N \\ F_N = mg\cos\beta \end{cases} \tag{S22}$$

Then, friction coefficient $\mu(\mu_-, \mu_+)$=$\tan\beta = \tan\left(\arctan\left(\frac{h}{\sqrt{l^2-h^2}}\right)\right)$. l and h are the length and height of inclined surface. These coefficients $\mu(\mu_-, \mu_+)$ are measured via the method depicted in **Fig. 6a**. **Fig. 6b** shows the steady-state displacement of the seta's contact point in the X-direction, validating the motion principles. Additional simulation data and parameters are provided in **Fig. 6c-e** and **Table 3**.

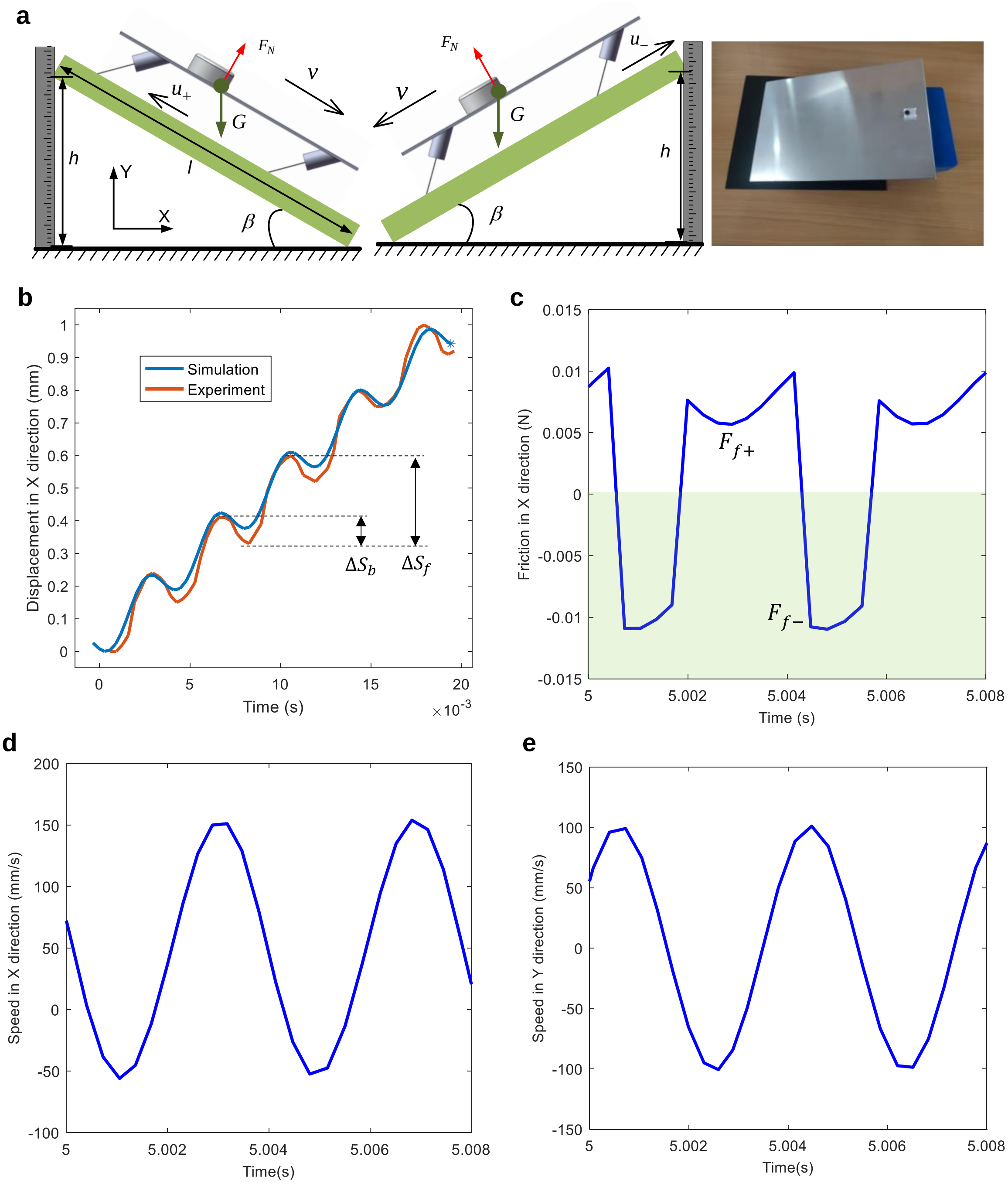

Fig. 6. Motion simulation results. (a) Friction coefficient measurement methodology. **(b)** Five-cycle X-direction displacement: simulation vs. experiment (Supplementary **Movie 1**). **(c)** Forward motion friction analysis. **(d)** X-direction speed profile. **(e)** Y-direction speed profile.

Table 3. Simulation Parameters Based on Cosserat Theory

k_i (N/m)	24.5/4	μ_+	0.5773
v_i	0.0316011/4	μ_-	0.7536
m (kg)	0.83×10^{-3}	γ	1.4
g (N/kg)	9.8	L (m)	0.005
k_i'	0.006/4	E (Pa)	7.3×10^{10}
v_i'	0.03/4	G (Pa)	2.81×10^{10}
d (mm)	0.1	ρ (g/cm^3)	6.5
θ	45°	A (m^2)	7.854×10^{-9}
$\mathbf{J}$ (m^4)	$\begin{bmatrix} 4.91e^{-18} & 0 & 0 \\ 0 & 4.91e^{-18} & 0 \\ 0 & 0 & 9.82e^{-18} \end{bmatrix}$	$\mathbf{K_{se}}$ (N)	$\begin{bmatrix} 220.5 & 0 & 0 \\ 0 & 220.5 & 0 \\ 0 & 0 & 573.3 \end{bmatrix}$
$\mathbf{K_{bt}}$ (Nm^2)	$\begin{bmatrix} 3.58e^{-7} & 0 & 0 \\ 0 & 3.58e^{-7} & 0 \\ 0 & 0 & 2.76e^{-7} \end{bmatrix}$	$\mathbf{B_{se}}$ (Ns)	$0_{3\times3}$
$\mathbf{C}$	$0_{3\times3}$	$\mathbf{B_{bt}}$	$0_{3\times3}$
ϱ	-0.48	c_1	-6.51×10^3
c_0	6.39×10^3	c_2	125.3
c_1	-6.51×10^3	d_1	-0.9231

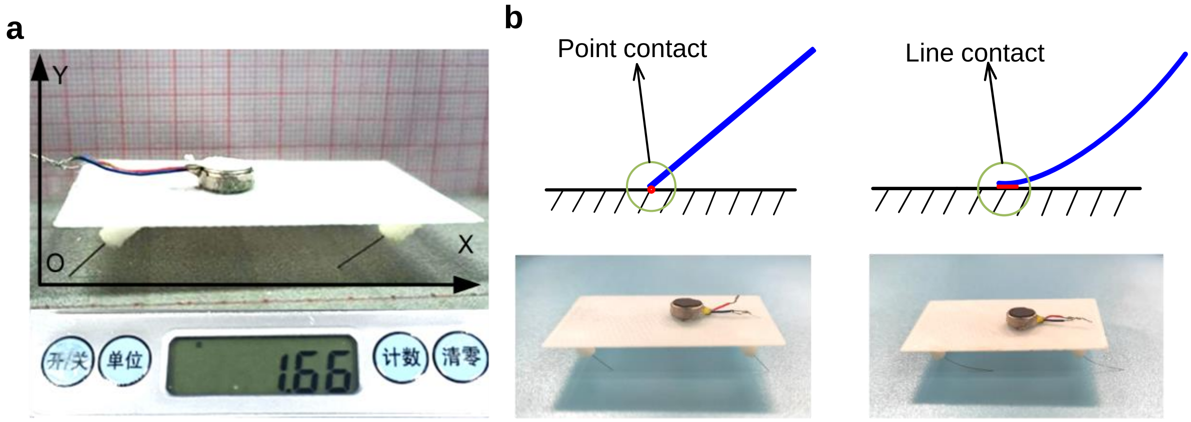

Fig. 7. FEbot prototype (5×3 cm) and seta-ground contact mechanics. **(a)** Robot weight characterization. **(b)** Friction variation with increasing seta length.

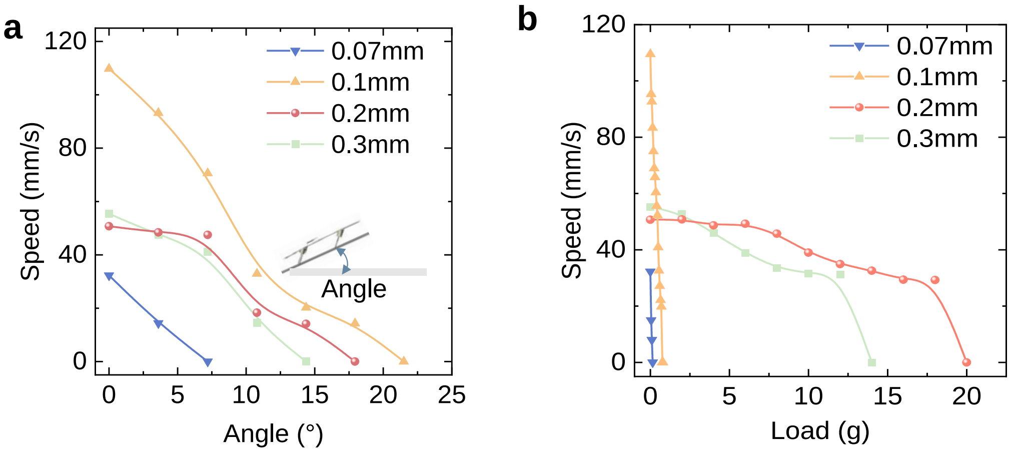

Fig. 8. Performance characterization. **(a)** Forward speed versus climbing angle for seta lengths = 7 mm, diameters = 0.07–0.3 mm, and angle = 60°. **(b)** Relative speed versus payload for seta diameters = 0.07–0.3 mm.

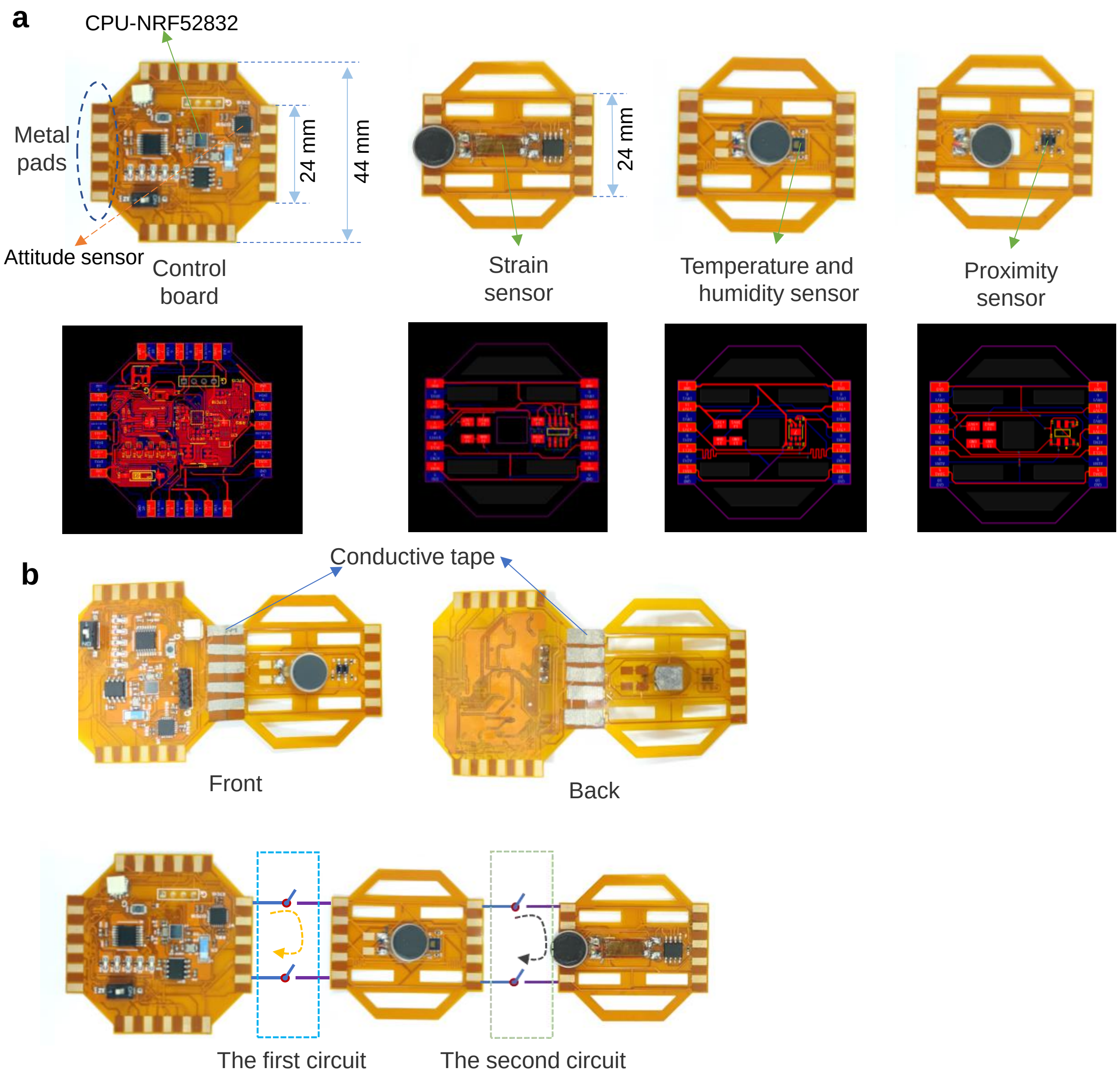

Fig. 9. Fabrication of modular components. **(a)** Four flexible electronics modules. **(b)** Assembly method for different types of FEbots.

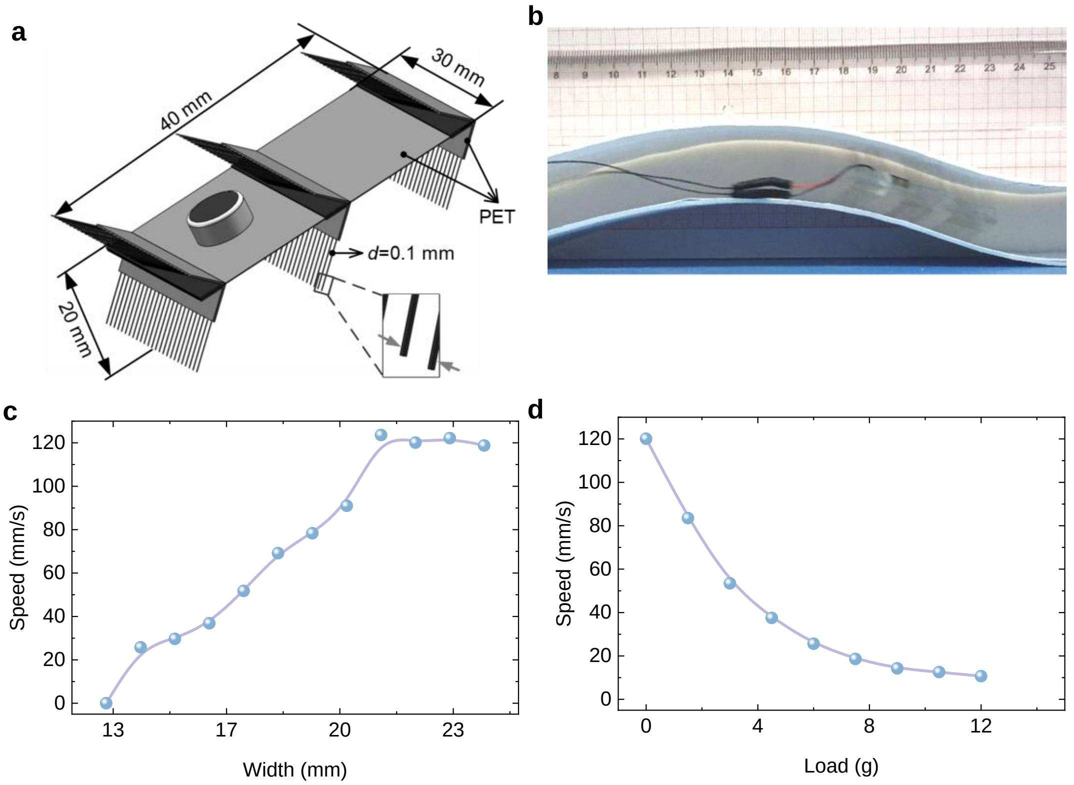

Fig. 10. Dual-sided FEbot with setae and performance characterization. **(a)** FEbot assembled from a setae module (polyethylene terephthalate (PET) film + SSMA) and an actuator module (oscillator + PET film). **(b)** Pathway on a horizontal surface. **(c)** Relative speed versus pathway width. **(d)** Load capacity versus relative speed.

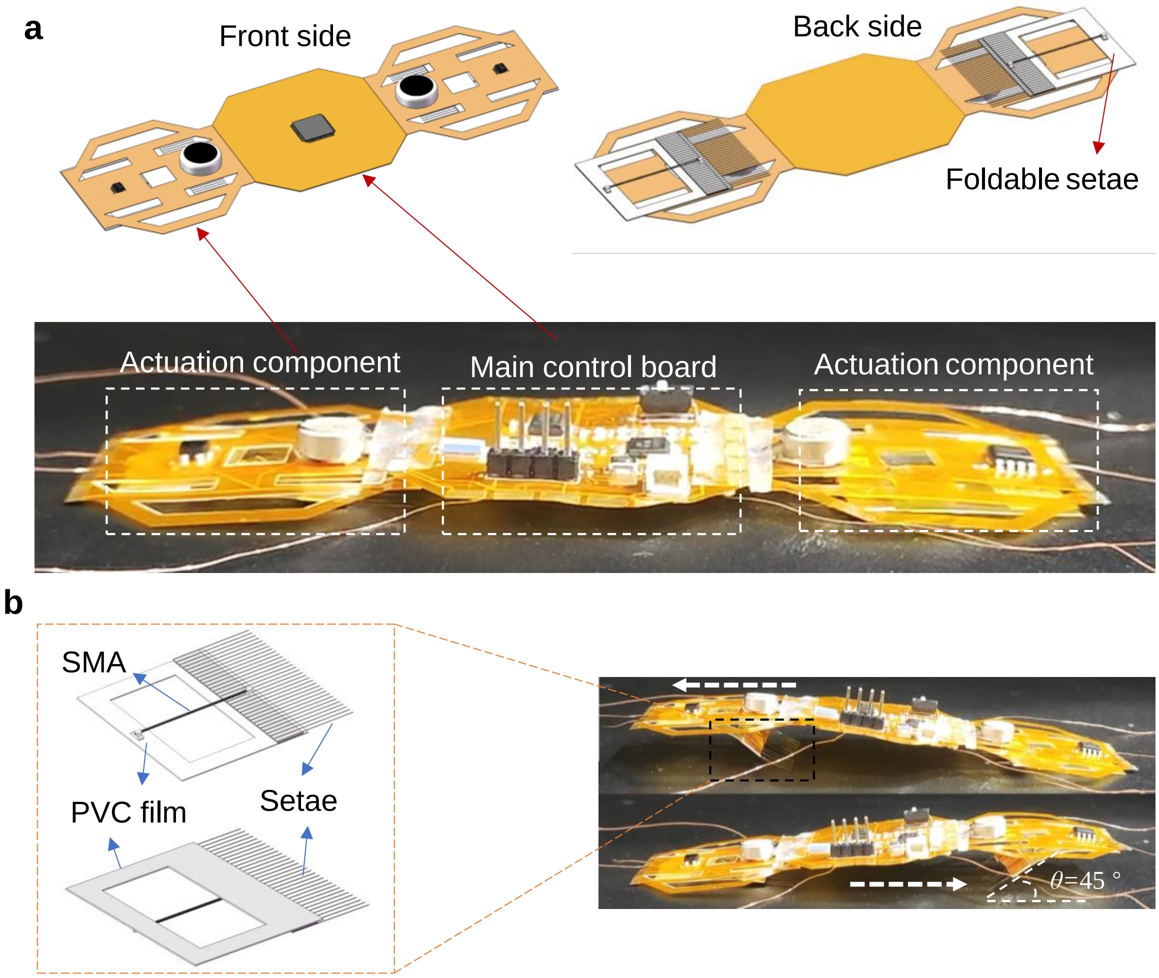

Fig. 11. Programmable assembly FEbot design. **(a)** Structure with foldable setae. **(b)** Achieving anisotropic friction via foldable setae.

Table 4 Programmable-Assembly FEbots and Locomotion Modes

Assemble type	Robot modules combined method					Robot function	Example
	Actuator and sensing module	Setae module					
**1**	a	1	2	3	4	Unidirectional motion with fastest speed and largest output force	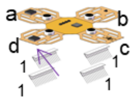 Unidirectional motion in x(+) direction
	b	1	2	3	4		
	c	1	2	3	4		
	d	1	2	3	4		
**2**	a	4	2	4	2	Bilateral rotation	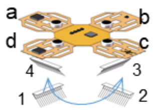 Both b and d actuated, or both a and c actuated
	b	3	1	3	3		
	c	2	4	4	2		
	d	1	3	3	3		
**3**	a	4	2	1	1	Unidirectional rotation, Movement along x or y directions	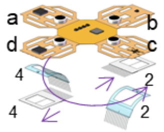 Unidirectional rotation, Turn left and turn right
	b	2	2	1	3		
	c	2	4	3	3		
	d	4	4	3	1		
**4**	a	2	4	1	1	Bilateral rotation, Movement along x or y directions	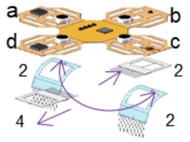 Bilateral rotation, Turn left and turn right
	b	2	4	1	3		
	c	2	4	3	3		
	d	4	2	1	3		

Note:

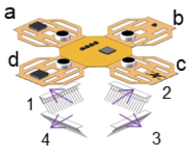
Omnidirectional translation

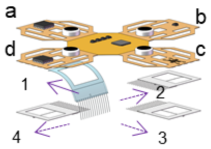
Omnidirectional translation

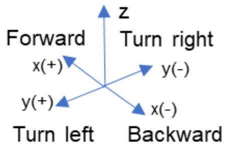

Coordinate System for movement

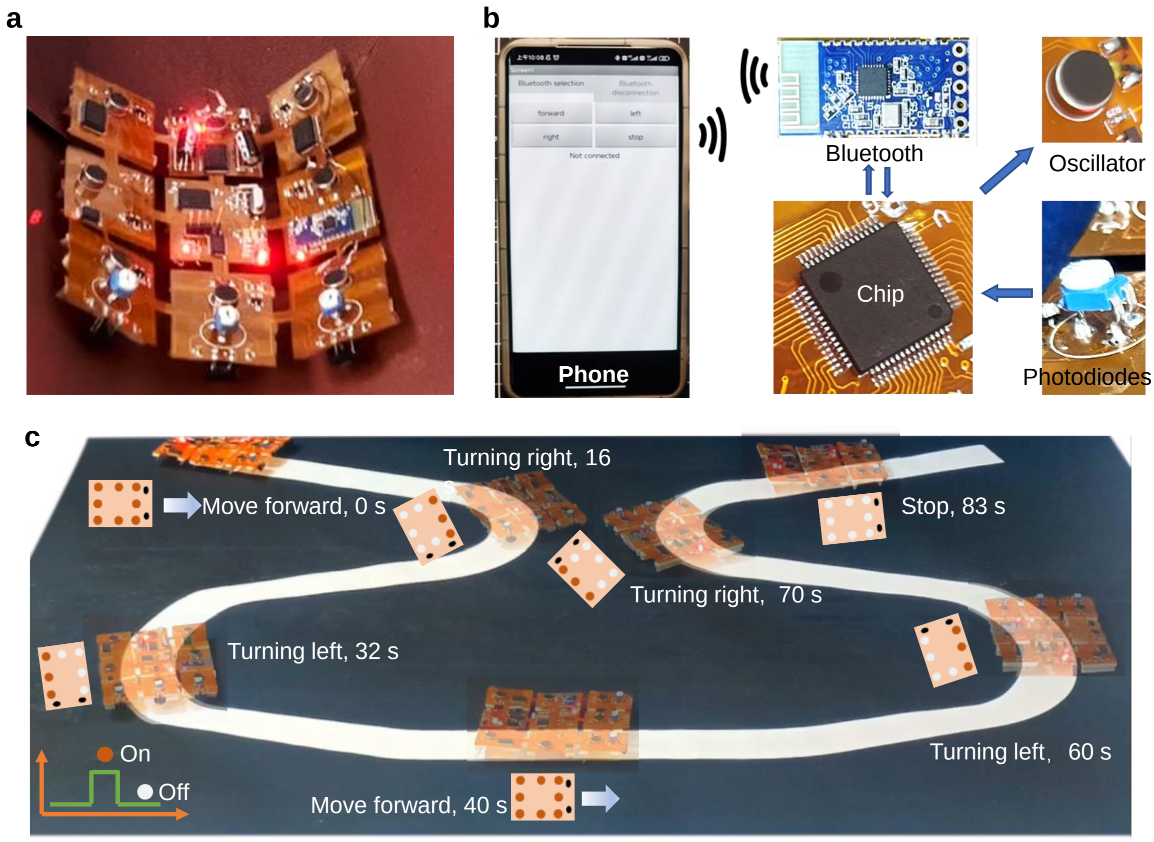

Fig. 12. Square-shaped FEbot (Type II). (a) Prototype photo. **(b)** Motion principle and snapshots of autonomous navigation through a Ω-shaped pathway. **(c)** Bluetooth remote control system.

Note 3. Calibration of strain sensors.

Fig. 13a illustrates the experimental setup for sensor calibration. The film embedded with integrated sensors is stretched incrementally at an elongation rate of 0.015 mm per second. The strain is defined as:

$$\varepsilon = \frac{l_1 - l_0}{l_0} \tag{23}$$

where l_0 and l_1 represent the initial and elongated lengths of the sensor, respectively. The output voltage between Pins 1 and 3 is calculated as:

$$V_{out} = \left(\frac{r_1}{r_1+r_2} - \frac{r_4}{r_3+r_4}\right)V_{in} \tag{24}$$

where r_1, r_2, r_3, r_4 denote the resistances of the sensor, and V_{in} denotes the input voltage applied between Pins 2 and 4. A Keithley DAQ6510 data acquisition card records V_{out} when V_{in}=10 V. As shown in **Fig. 13b**, the experimental data exhibit a strong linear relationship between strain and voltage variation.

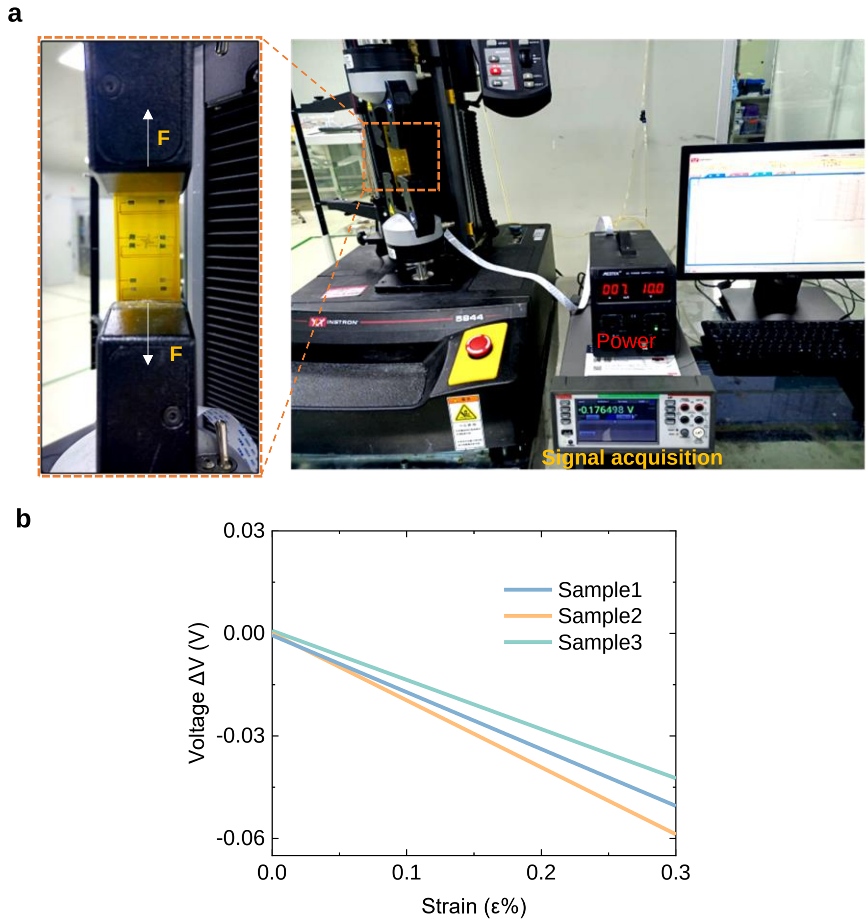

Fig. 13. Strain sensor calibration. **(a)** Experimental setup. **(b)** Strain *versus* voltage variation.

Note 4. Shape reconstruction method.

To achieve shape reconstruction, the curvature of the FEbot must first be calculated. As shown in **Fig. 14**, the geometric equation for an infinitesimal element within an arbitrary segment can be expressed as:

$$\begin{cases} \left(\chi + \frac{\delta}{2}\right)\vartheta = Q + \Delta Q \\ \left(\chi - \frac{\delta}{2}\right)\vartheta = Q - \Delta Q \\ \varepsilon = \Delta Q / Q \end{cases} \tag{25}$$

where δ and Q represent the thickness and length of the infinitesimal element, respectively; ΔQ is the variation in length after deformation; χ denotes the length of the central layer, which remains unchanged post-deformation; and ϑ is the bending angle. The curvature is therefore given by:

$$\kappa = \frac{1}{\chi} = \frac{2\varepsilon}{\delta} \tag{26}$$

Fig. 15a illustrates the shape reconstruction method based on the FEbot's curvature in its static state and the homogeneous transformation matrix computed via a Denavit-Hartenberg (D-H) approach. The soft body is divided into multiple segments using a linear interpolation algorithm, driven by strain data from three embedded sensors. The curvature for each segment can be rewritten as:

$$\kappa_\varsigma = \frac{1}{\chi} = \frac{2\varepsilon_\varsigma}{\delta} \tag{27}$$

where ε_ς is the strain of the ς-th segment ($\varsigma = 1, 2, \cdots, n$).

The included angle α_ς between the line segment $P_\varsigma P_{\varsigma+1}$ and the axis b_ς is defined as:

$$\alpha_\varsigma = \varphi_\varsigma ds \tag{28}$$

where P_ς is the origin of the local coordinate system $M_\varsigma(a_\varsigma, b_\varsigma)$, and $ds = \Phi/\xi$ represents the length of an arbitrary segment, with Φ denoting the distance between the centers of adjacent sensors.

The transformation matrix from the local coordinate system $M_\varsigma(a_\varsigma, b_\varsigma)$ to $M_{\varsigma+1}(a_{\varsigma+1}, b_{\varsigma+1})$ is given by:

$$\Psi_{\varsigma+1} = \begin{bmatrix} 1 & 0 & 0 & ds\cdot\cos(\alpha_\varsigma) \\ 0 & 1 & 0 & ds\cdot\sin(\alpha_\varsigma) \\ 0 & 0 & 1 & 0 \\ 0 & 0 & 0 & 1 \end{bmatrix} \begin{bmatrix} \cos(-\alpha_\varsigma) & -\sin(-\alpha_\varsigma) & 0 & 0 \\ \sin(-\alpha_\varsigma) & \cos(\alpha_\varsigma) & 0 & 0 \\ 0 & 0 & 1 & 0 \\ 0 & 0 & 0 & 1 \end{bmatrix} \tag{29}$$

while the transformation matrix from $M_{\varsigma+1}$ to the global coordinate $\boldsymbol{\Gamma}$ is expressed as:

$$\Lambda = \begin{bmatrix} 1 & 0 & 0 & 0 \\ 0 & 1 & 0 & 0 \\ 0 & 0 & 1 & 0 \\ 0 & 0 & 0 & 1 \end{bmatrix} {\Psi^{-1}}_2 {\Psi^{-1}}_3 \cdots {\Psi^{-1}}_{\varsigma+1} M_{\varsigma+1} \tag{30}$$

Fig. 15b demonstrates the shape reconstruction results for varying arc radii (η).

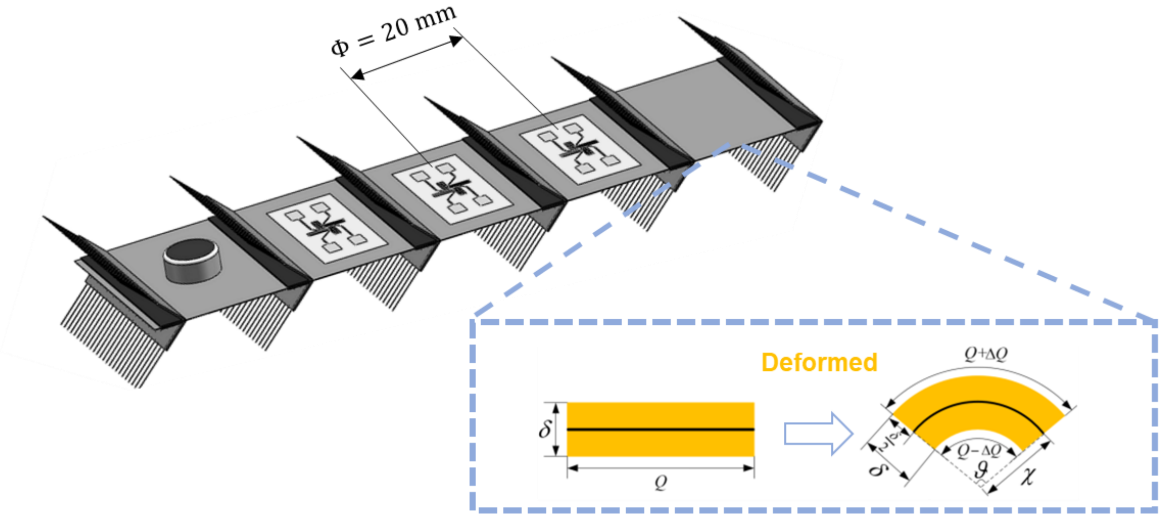

Fig. 14. Deformation analysis of an infinitesimal element.

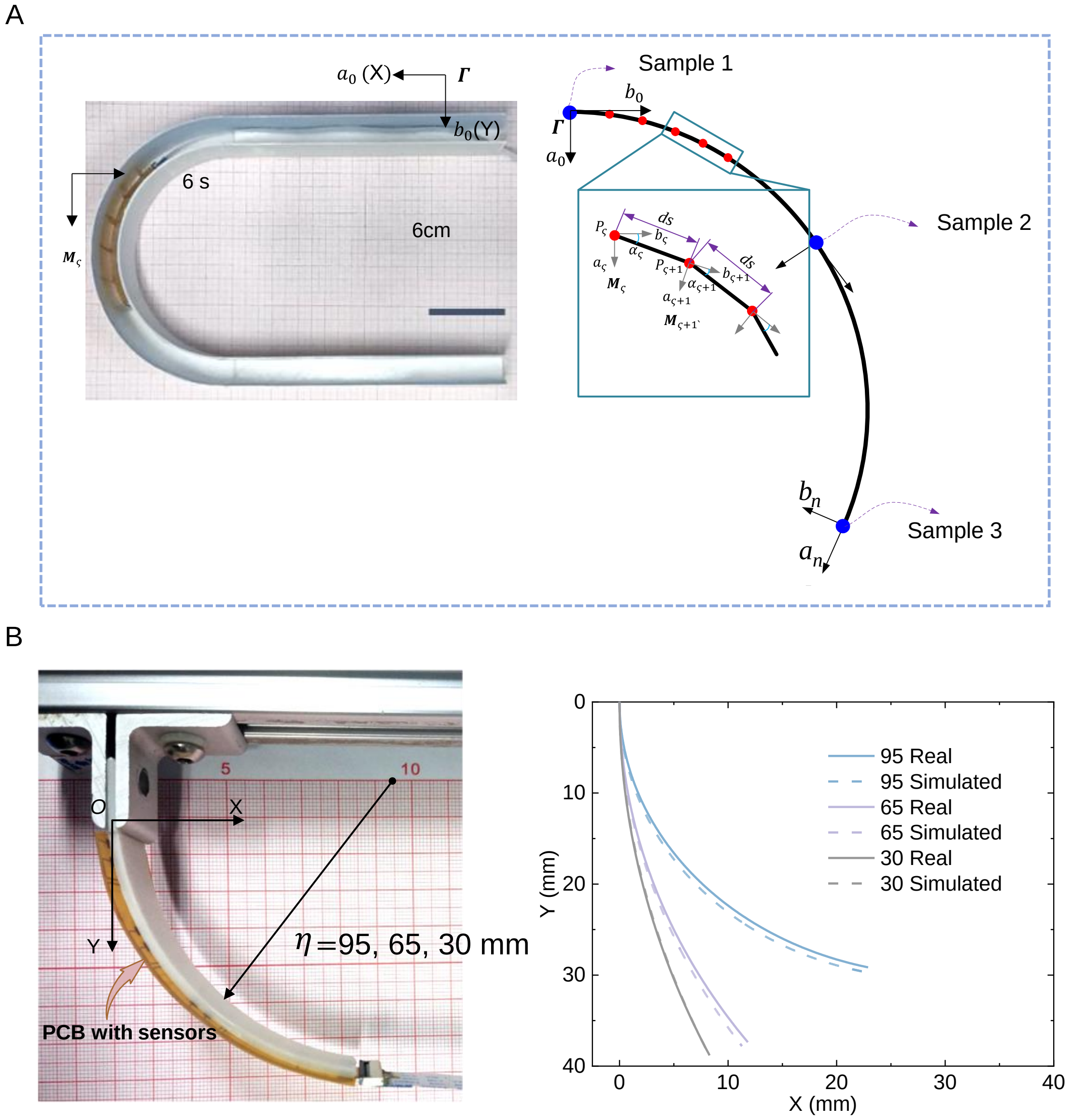

Fig. 15. Shape reconstruction methodology. **(a)** Homogeneous transformation matrix computed via D-H-type approach. **(b)** Real *versus* simulated shape comparison.

As shown in **Fig. 16**, the FEbot acquires strain data from time Z_0 to Z_{j+1}. The curvature matrix for the three sensors over this time interval is defined as:

$$\begin{cases} \varkappa_1 = [\varphi_{10}, \cdots, \varphi_{1j}, \varphi_{1(j+1)}] \\ \varkappa_2 = [\varphi_{20}, \cdots, \varphi_{2j}, \varphi_{2(j+1)}] \\ \varkappa_3 = [\varphi_{30}, \cdots, \varphi_{3j}, \varphi_{3(j+1)}] \end{cases} \tag{S31}$$

Theoretically, the relationships between curvature values can be derived as:

$$\begin{cases} \varphi_{2(j+1)} = \varphi_{1j} \\ \varphi_{3(j+1)} = \varphi_{2j} \end{cases} \tag{S32}$$

Using the curvature matrix $[\varphi_{1j}, \cdots, \varphi_{2j}, \varphi_{3j}]$, the robot's shape at time Z_j can be reconstructed via Eqs. (25–S30). The entire pathway geometry is then obtained by sequentially connecting the robot's reconstructed shapes through overlapping regions. However, Eq. (32) introduces inaccuracies in practice due to cumulative errors from sensor noise and robot assembly imperfections. To address this, a simplified method is proposed, leveraging strain measurements from a single sensor. For example, 27 discrete points are selected from the 147 original data points (measured using a Keithley DAQ6510) to construct the curvature matrix C_1. The selection interval is determined by $\Phi/(v_{Febot})$, where v_{Febot} is the FEbot's average moving speed.

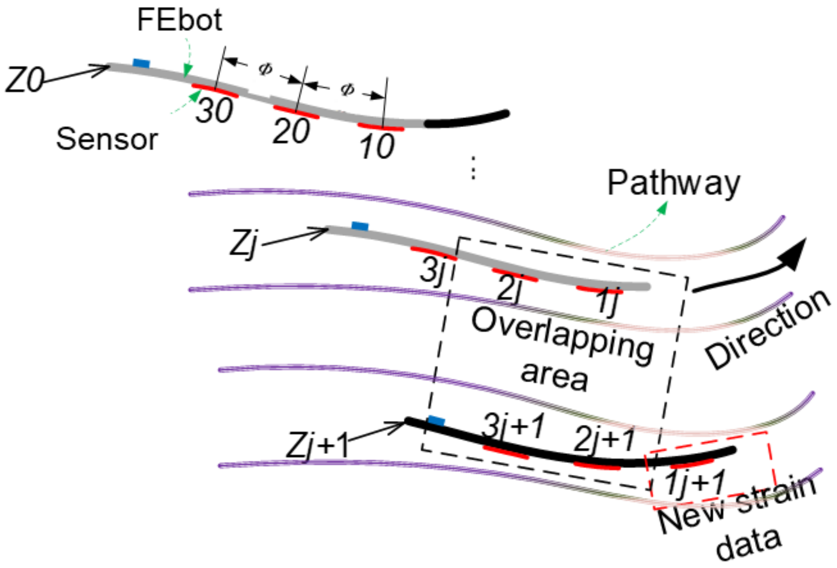

Fig. 16. Curve fitting method.

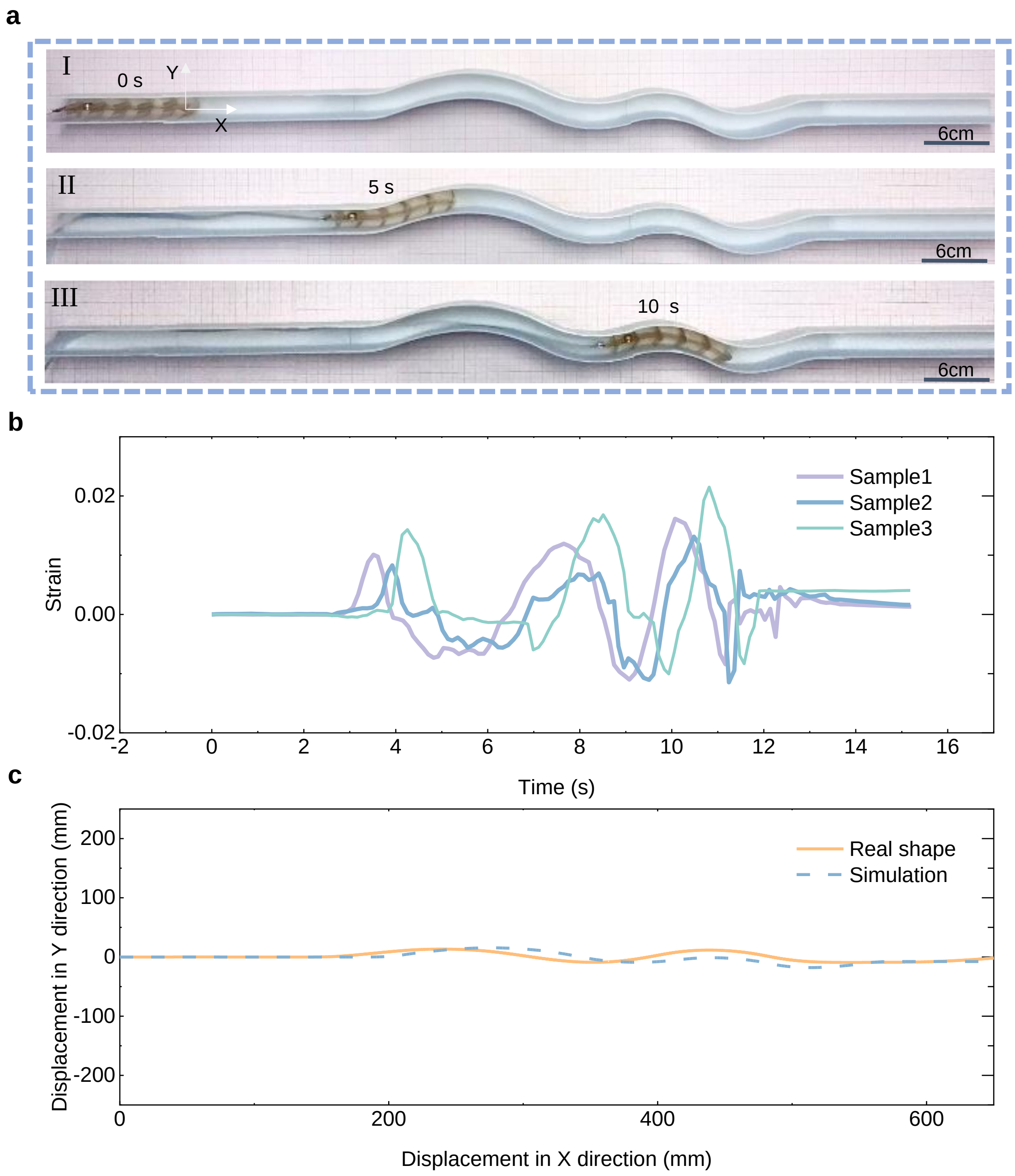

Fig. 17. "S"-shaped pathway shape recognition. **(a)** Strain data acquisition experiment. **(b)** Measured sensor strain. **(c)** Reconstructed vs. actual shape.

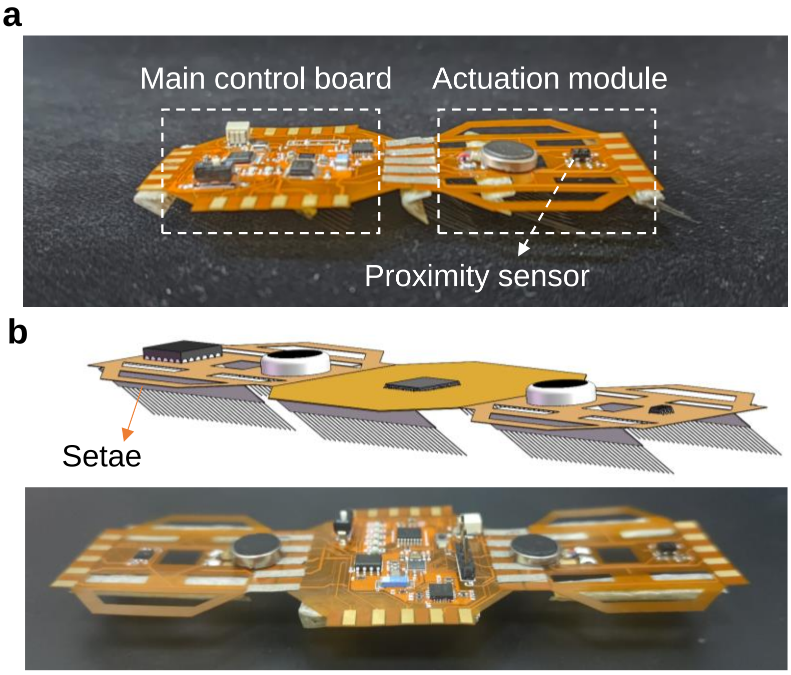

Fig. 18. Design and control of FEbots. **(a)** Structure with control board, actuation module, and proximity sensor. **(b)** Steering-capable FEbot.

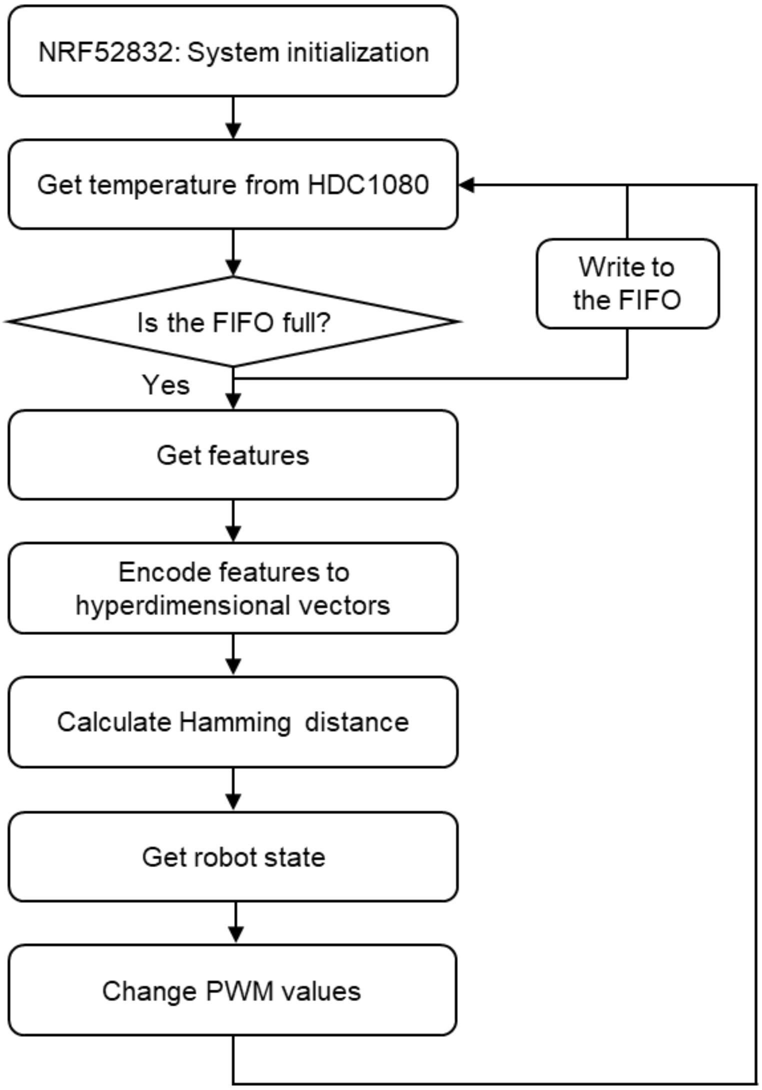

Fig. 19. Closed-loop temperature tracking control.

NRF52832 initializes, then collects temperature data from the HDC1080 sensor, extracts and encodes feature values according to the first in first out (FIFO) principle, calculates the Hamming distance to determine the robot status, and consequently controls the Pulse Width Modulation (PWM) output.

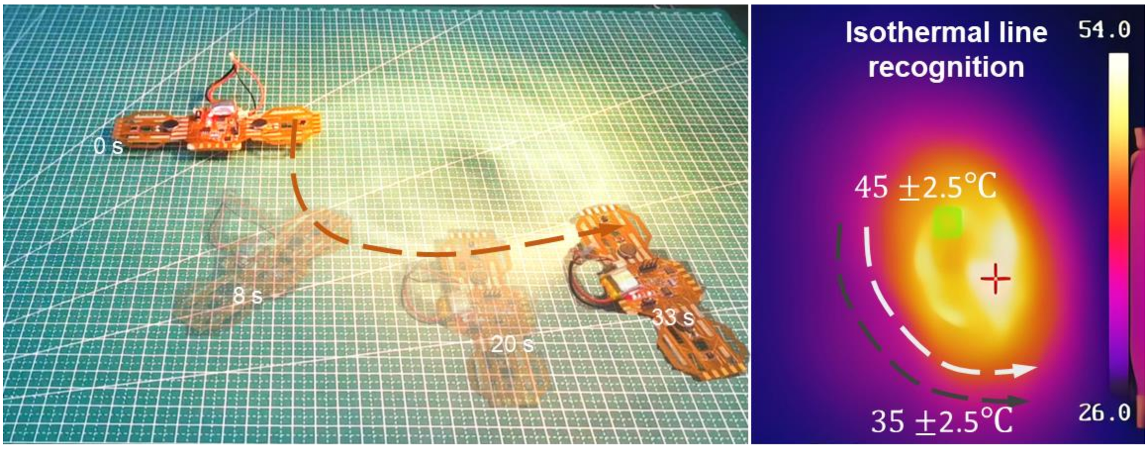

Fig. 20. AI-embodied FEbot for isothermal line recognition.

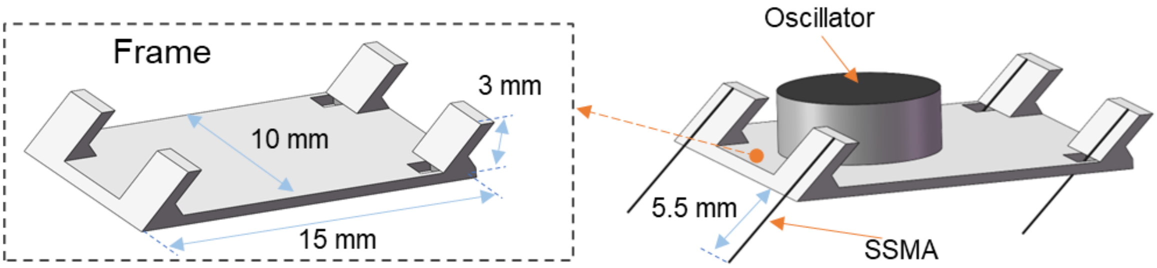

Fig. 21. Single-unit FEbot design.

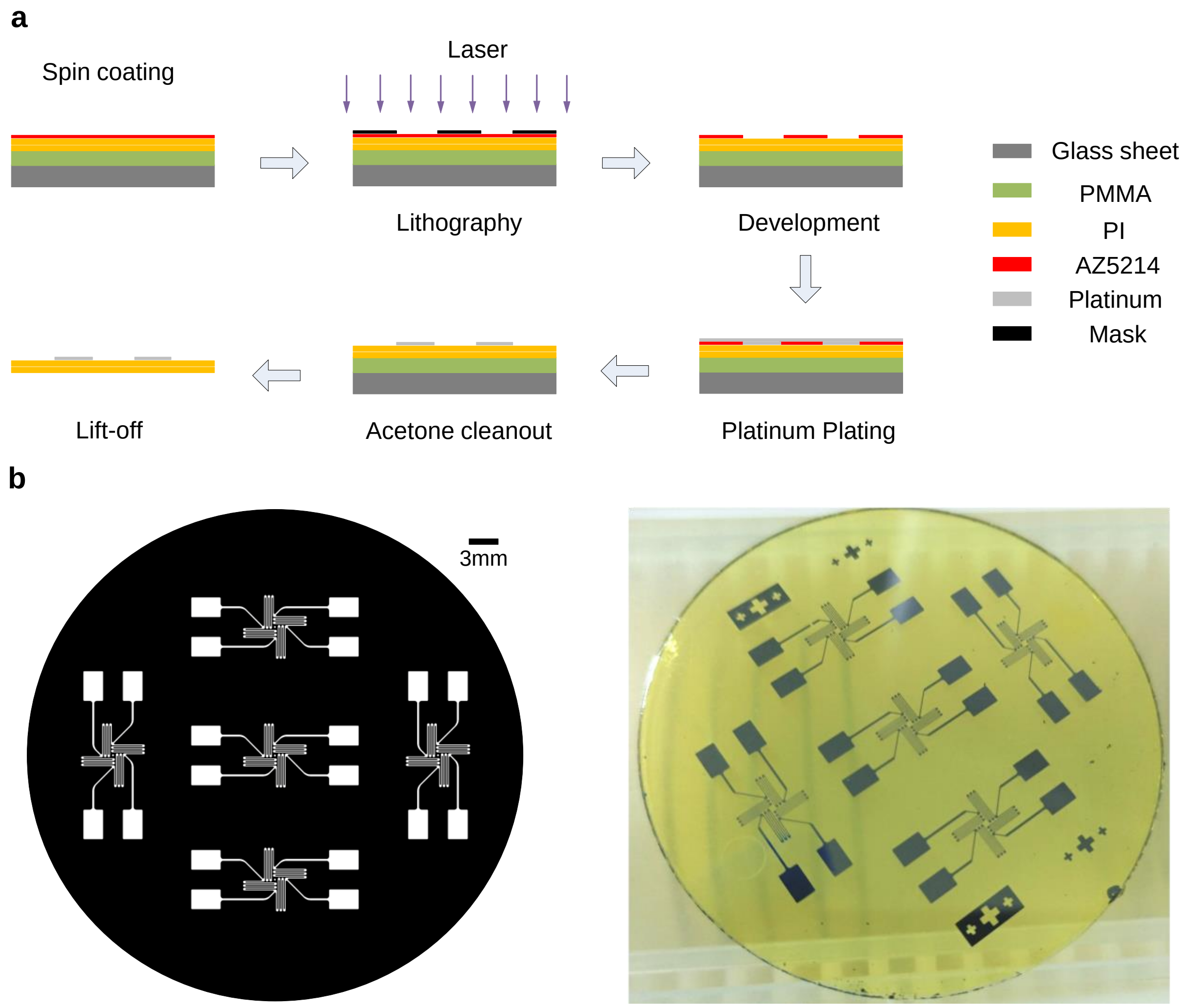

Fig. 22: Strain sensor fabrication. **(a)** Fabrication process. **(b)** Sensor mask design and fabricated samples.

Table 5 Fabrication Process Parameters

Material	Low speed (r/min) ×time (s)	High speed (r/min) ×time (s)	Acceleration (r/min^2)	Deceleration (r/min^2)	Heating method
PMMA	500×15 s	3000×60 s	500	800	Hot plate: 180℃, 5min。
PI		2000×90 s	500	800	Hot plate: 180℃. 5min; Baking oven 220℃, >4 hours。
AZ5214	500×15s	3000×60 s	500	800	Hot plate: 95 ℃, 60 s。
