# Supplementary material for: AI-embodied multi-modal flexible electronic robots with programmable sensing, actuating and self-learning
Source: Nat Commun. 2025 Oct 3;16:8818. doi: 10.1038/s41467-025-63881-6 (PMC12494961; doi:10.1038/s41467-025-63881-6)
Supplement: Supplementary file 2 — Description of Additional Supplementary Files [file 41467_2025_63881_MOESM2_ESM.pdf]

Description of Additional Supplementary Files

Supplementary Code 1 - Code for locomotion simulation

Supplementary Movies

Supplementary Movie 1: Video taken with a high-speed camera showing the locomotion Supplementary Movie 2: A unit of FEbot running at the fastest speed of 109.5 mm/s (2.19 BL/s) Supplementary Movie 3: Performance of unit attached load

Supplementary Movie 4: A millipede-like FEbot (Type I) climbing a vertical pathway

Supplementary Movie 5: Performance of foldable setae modules

Supplementary Movie 6: Moving forward and backward in confined space based on foldable setae modules

Supplementary Movie 7: Weight-bearing test for FEbot (Type I)

Supplementary Movie 8: A square-shape FEbot (type II) achieving movement in different directions Supplementary Movie 9: A square-shape FEbot (type II) crawling in smooth pipe with folding shape Supplementary Movie 10: Autonomously navigating a figure Ω for FEbot (Type II)

Supplementary Movie 11: Underwater straight-line crawling and turning of a square-shape FEbot (type II)

Supplementary Movie 12: A square-shape FEbot (type II) crawling step even after turning over

Supplementary Movie 13: Multi-modal perception for FEbot (Type I) when it goes through the S-shaped pathway

Supplementary Movie 14: A real-time video for environmental surveillance

Supplementary Movie 15: Shape recognition of the “U”-shaped and “S”- shaped pathway Supplementary Movie 16: AI-embodied FEbot capable of autonomous escaping danger

Supplementary Movie 17: AI-embodied FEbot capable of autonomous identification a temperature field and achieving obstacle avoidance
